# Detection of Honey Adulteration Using UV–Vis Absorption Spectroscopy and Chemometrics

**DOI:** 10.3390/foods15172953

**Published:** 2026-08-22

**Authors:** Aya Ibrahim, Mohamed O. Amin, Bessy D’Cruz, Bhavik Vyas, Igor K. Lednev, Entesar Al-Hetlani

**Affiliations:** 1 Department of Chemistry, College of Science, Kuwait University, Sabah Al Salem University City, P.O. Box 5969, Safat 13060, Shadadiya, Kuwait; s2201130006@sci.ku.edu.kw (A.I.); bessy.dcruz@ku.edu.kw (B.D.); 2 Department of Chemistry, University at Albany, State University of New York, 1400 Washington Avenue, Albany, NY 12222, USA; moamin@albany.edu (M.O.A.); bvyas@albany.edu (B.V.)

**Keywords:** honey authentication, honey adulteration, sugar syrups, food fraud, UV–vis spectroscopy, chemometrics

## Abstract

This preliminary study demonstrates the use of ultraviolet–visible (UV–vis) absorption spectroscopy coupled with chemometric modeling to detect and quantify honey adulteration. UV–vis spectra of pure honey and samples experimentally adulterated with corn, agave, and date syrups were collected within the 200–500 nm range after their simple dilution with water. Orthogonal partial least squares discriminant analysis (OPLS-DA) distinguished pure honey from adulterated samples, achieving 88% accuracy on an external validation dataset. A second set of classification models differentiated between honey containing a single adulterant and that containing multiple adulterants, with a high prediction rate based on an external validation dataset. Quantitative analysis of adulterant content via orthogonal partial least squares regression analysis revealed the strong predictive performance of the models (R^2^ ≥ 0.87) on an external validation dataset. The proposed proof-of-concept study provides a rapid, cost-effective, and non-destructive screening tool for honey authentication with minimal sample preparation.

## 1. Introduction

Olive oil, milk, honey, saffron, orange juice, coffee, and apple juice are among the foods most frequently adulterated for economic gain [1]. Among these, honey holds religious and cultural importance worldwide and stands out as a naturally produced organic sweetener that can be consumed without extensive processing [2]. Additionally, its therapeutic properties have long been recognized in traditional medicine, where it is employed to treat various health conditions, including respiratory infections, gastrointestinal disorders, skin ailments, and wounds [3].

In Kuwait and across the Middle East, *Ziziphus spina-christi* (Sidr) honey is highly valued for its distinctive flavor, medicinal properties, and perceived purity [4,5]. Honey production in Kuwait is concentrated in agriculturally rich regions such as Wafra in the south (S) and Abdali in the north (N), where Sidr honey constitutes the primary harvest, complemented by smaller seasonal yields of eucalyptus and willow honeys [6]. Sidr honey commands premium prices in local and international markets owing to its unique characteristics and enjoys global recognition, ranking close behind Manuka honey in popularity [7]. However, growing demand for Sidr honey and its high commercial value have made it a prime target for falsification and adulteration, threatening consumer confidence and producer reputation. Detecting such adulteration is essential for safeguarding the integrity of the honey industry and ensuring product authenticity [8].

Honey is frequently adulterated with inexpensive sweeteners such as refined cane and beet sugars and high-fructose corn and maltose syrups to maximize commercial profits. Adulteration can occur directly, through the addition of sweeteners or blending low-quality honey with premium-grade varieties, and indirectly, through feeding bees substances such as sugar syrups, antibiotics, or pesticides, making its detection challenging via routine analytical techniques [9,10]. Consequently, numerous studies have focused on developing robust methodologies for honey authentication utilizing a wide array of advanced analytical techniques. Among these, stable carbon isotope ratio analysis and liquid chromatography–isotope ratio mass spectrometry (LC-IRMS) are widely recognized as the gold standard for detecting adulterants and distinguishing honeys of varying botanical origin [11,12]. Although highly effective for honey authenticity assessment, the sensitivity of this technique may vary depending on the type and level of adulteration, particularly in certain low-level adulteration scenarios [12,13]. In addition, proton nuclear magnetic resonance (^1^H NMR) spectroscopy has gained considerable attention as one of the most comprehensive approaches for honey authentication, enabling the simultaneous characterization and quantification of multiple honey constituents and the detection of adulterants such as sugar syrups and exogenous sugars. Despite its excellent repeatability, rapid analysis, and minimal sample preparation requirements, the widespread application of NMR remains limited by its high acquisition and maintenance costs, lack of portability, and the need for highly skilled operators [14]. Likewise, chromatographic and mass spectrometric techniques (e.g., quadrupole time-of-flight mass spectrometry [15], high-performance anion-exchange chromatography [16], gas chromatography [17], and high-performance liquid chromatography [18,19]) offer high sensitivity, accuracy and specificity for honey authentication. However, these analytical approaches often require extensive sample preparation, costly instrumentation, longer analysis times, and specialized expertise for data interpretation, which may limit their suitability for rapid routine screening or portable field-based applications. Consequently, spectroscopic techniques integrated with advanced statistical analysis have attracted considerable attention as cost-effective screening tools for detecting honey adulteration. Among them, infrared [20], near-infrared (NIR) [9], and Raman [21] spectroscopies have proven highly effective in assessing honey authenticity. These spectroscopic approaches offer notable advantages, including rapid sample analysis, non-destructive testing, user-friendly operation, and suitability for real-world applications, making them particularly attractive for routine quality assurance and food fraud prevention.

Nevertheless, ultraviolet–visible (UV–Vis) absorption spectroscopy has received relatively limited attention in honey adulteration studies despite its potential as a simple, cost-effective, environmentally friendly, and non-destructive analytical technique. UV–Vis spectroscopy requires relatively small sample volumes and preserves sample integrity, allowing subsequent analysis by complementary techniques when necessary [22]. Recent research indicates that combining UV–vis spectroscopy with chemometric modeling substantially enhances the ability to detect adulterants. For example, UV–vis spectroscopy combined with advanced chemometric models such as one-class partial least squares (OC-PLS) and data-driven soft independent modeling of class analogy (DD-SIMCA) demonstrated high accuracy in detecting honey adulterated with high-fructose corn and agave syrups and sugarcane molasses [23]. Razavi and Kenari [22] coupled UV–vis absorption spectroscopy with machine learning (ML) algorithms to evaluate the sucrose content of authentic (n = 12), commercial (n = 12), and adulterated (n = 16) honey samples. Correlation analysis between sucrose concentration and UV–vis absorbance identified four discriminative wavelengths at 216, 280, 316, and 603 nm. Among the tested ML models, the support vector regression (SVR) model yielded the most accurate calibration (root mean square error [RMSE] = 0.97 and R^2^ = 0.98), outperforming the partial least squares regression (PLSR) model. Similarly, a multivariate chemometric approach was successfully coupled with UV–vis spectroscopy to distinguish pure Mediterranean thyme honey from samples adulterated with sugar syrups or colorants, whereby principal component analysis (PCA) was employed in the initial exploratory data analysis and supervised classification models (e.g., random forest (RF), PLS-DA, and DD-SIMCA) were utilized to accurately discriminate between authentic and adulterated honey [24]. PCA was also applied to differentiate between pure Brazilian polyfloral honey and samples adulterated with glucose syrup [25]. Moreover, a data fusion strategy combining UV–vis and NIR spectroscopies with PLS modeling and artificial neural networks (ANNs) improved predictions of the physical and chemical properties of honey adulterated with varying concentrations (10–90%) of corn syrup [26]. In a related study, UV–Vis spectroscopy combined with an artificial neural network model was used to detect and quantify adulteration in six Romanian honey types mixed with commercial syrups at concentrations between 5% and 50%. The model accurately identified adulteration regardless of sample type and reliably detected levels above 10%, highlighting its potential as a rapid and cost effective tool for honey authenticity assessment [27].

Herein, UV–Vis absorption spectroscopy coupled with machine learning was investigated for the qualitative and quantitative assessment of adulteration in Kuwaiti Sidr honey. Despite the high commercial value and regional importance of Kuwaiti Sidr honey, studies examining its authenticity using rapid spectroscopic approaches remain limited. To address this gap, the present study extends previous investigations by evaluating a larger sample set and a broader range of commercially relevant adulterants, including both single- and multiple-adulterant systems. Specifically, Kuwaiti Sidr honey was adulterated with corn, date, and agave syrups at varying concentrations, individually and in combination, to generate binary, ternary, and quaternary mixtures. The study further integrates orthogonal partial least squares discriminant analysis (OPLS-DA) for qualitative classification with orthogonal partial least squares regression (OPLS-R) for quantitative estimation of adulterant levels, enabling both detection and quantification of adulteration across diverse adulteration scenarios. To our knowledge, this is the first UV–Vis spectroscopic study of Kuwaiti Sidr honey to simultaneously evaluate single- and multiple-adulterant systems while integrating OPLS-based qualitative classification and quantitative prediction models. The proposed approach is rapid, eco-friendly, non-destructive, and demonstrates strong potential for translation to portable or handheld devices, offering practical benefits for honey researchers, producers, exporters, retailers, regulatory agencies, and consumers.

## 2. Materials and Methods

### 2.1. Sample Collection

Eleven batches of Sidr honey produced between November 2023 and February 2024 were purchased from local supermarkets, retail shops, and farms located in the Abdili (northern) and Al-Wafra (southern) regions of Kuwait. Four jars were collected for each honey batch, yielding 44 pure Sidr honey samples. All honey samples were verified based on labeling and provenance information. Although batch numbers were not consistently available, production dates were provided, and all batches originated from the same harvest season to ensure comparability. The authenticity of the honey samples was confirmed through our previous experimental characterization of the same honey lots, including physicochemical analysis, elemental analysis, and LC–MS-based sugar profiling [6,28]. All measured parameters were consistent with those reported for authentic Kuwaiti Sidr honey and fell within the expected compositional ranges. Table S1 presents the honey symbols, corresponding sources, and dollar values. For the adulteration experiments, corn, agave, and date syrups were obtained from local supermarkets and employed as representative model adulterants.

The study analyzed 1100 honey samples, comprising 44 pure Sidr honey samples and 1056 samples experimentally adulterated with corn, agave, and/or date syrups in various combinations. Although the study included a large number of experimentally generated adulterated samples, the natural variability of the authentic honey was limited to 44 samples obtained from 11 commercially sourced Sidr honeys (Table 1). Future studies will expand the dataset to include authentic honey samples from a wider range of botanical and geographical origins, as well as commercially adulterated honey, to further evaluate the robustness and generalizability of the proposed approach. All honey and syrup samples were maintained in their original containers and stored in the dark at room temperature (20–25 °C) until analysis. In this study, no noticeable crystallization-related issues or significant spectral instability were observed during sample preparation, storage, or spectral acquisition. However, the effects of long-term storage and crystallization on the spectral characteristics and performance of the chemometric models were not systematically evaluated in the present study. Ultrapure deionized water (18.2 MΩ·cm), produced using a PURELAB Chorus 1 system (ELGA, High Wycombe, UK), was employed to dilute honey samples for UV–vis spectroscopic analysis. No filtration or centrifugation was performed prior to analysis.

### 2.2. Sample Treatment

Prior to UV–vis spectroscopic analysis, honey samples were liquefied in a water bath at 40 °C for 10 min and manually stirred to ensure homogenization. The concentration of the honey solutions was optimized to prevent saturation of the acquired absorption spectra. Aqueous stock solutions (30 μg/mL) of honey samples and adulterants (corn, date, and agave syrups) were prepared in 100 mL volumes.

Binary, ternary, and quaternary mixtures of pure honey and adulterants were prepared as previously described [23] and are outlined in Table 1. Binary mixtures were prepared with honey and corn syrup at concentrations ranging from 10% to 90% (*v*/*v*). Ternary mixtures were prepared with honey and corn and date syrups. Quaternary mixtures were prepared with honey and corn, date, and agave syrups. The adulterated honey samples were sonicated at room temperature for 60 min to ensure homogeneity before analysis.

### 2.3. Instrumentation, Data Preprocessing, and Statistical Analysis

UV–vis spectra were recorded using a quartz cuvette with an optical path length of 1 cm and a Cary 50 UV–Vis spectrophotometer (Varian, Palo Alto, CA, USA) equipped with a photodiode array detector covering the 190–1100 nm range, with a resolution of 1 nm. Measurements were performed at room temperature within the 200–500 nm range. Honey samples were measured in triplicate, yielding 12 spectra per sample (4 jars × spectral scans).

UV–vis spectra were imported into MATLAB R2024a (MathWorks, Inc., Natick, MA, USA) equipped with PLS_Toolbox version 9.5 (Eigenvector Research, Inc., Wenatchee, WA, USA) for data preprocessing and chemometric analysis. Spectral preprocessing was performed using a second-order Savitzky–Golay first derivative (window size of 11 points) to remove baseline variations and enhance subtle spectral features. Following preprocessing, PCA was applied to reduce data dimensionality and capture the maximum variance within the dataset.

Herein, adulteration was defined as the presence of any added adulterant in honey, independent of concentration. A hierarchical chemometric workflow was implemented to detect adulteration and quantify the adulterant content. First, an OPLS-DA model was trained to classify honey samples as pure versus adulterated. Second, a separate OPLS-DA model was constructed to discriminate honey containing a single adulterant from that containing multiple adulterants. Finally, OPLS-R analysis was performed to estimate the adulterant concentration in each sample as a continuous outcome, enabling quantitative assessment beyond binary detection.

Model performance was initially evaluated using an internal validation dataset with random subset cross-validation (RSCV), comprising 10 splits and 10 iterations. The predictive performance of the model was further assessed using an independent external validation dataset of honey samples that were not used during model training. As summarized in Table S1, the 11 independent Sidr honeys were randomly assigned to either the calibration or external validation set at the honey level, ensuring complete independence between model training and testing. Accordingly, once a honey source was assigned to the calibration dataset, all four jars and all spectra derived from that honey were included exclusively in the calibration dataset. Similarly, if a honey source was assigned to the external validation dataset, all four jars and all corresponding spectra remained exclusively in the external validation dataset. Consequently, no jars or spectra from the same honey source were shared between the calibration and external validation datasets, preventing any overlap of technical replicates between model training and independent testing. The training set comprised 24 pure honey samples and 576 adulterated samples, while the test set included 20 pure honey samples and 480 adulterated samples. In total, 1800 and 1500 spectra were employed for model training and independent validation, respectively. All honey samples were collected during the same harvest season to minimize seasonal variation. Although this reduced seasonal variability, future studies should include samples from multiple harvest seasons to further evaluate the robustness and generalizability of the developed models.

## 3. Results and Discussion

### 3.1. Spectral Analysis

One of the most common honey adulteration practices involves adding sugar syrup to increase its volume and profit margin [29]. Such adulteration can lead to considerable health risks and economic loss. Corn, agave, and date syrups are widely employed as commercial sweeteners and closely mimic the physicochemical properties of honey. Agave syrup, derived from agave plants, is particularly rich in fructose [30,31]. Corn syrup, produced from maize, is a primary source of glucose that is widely adopted in the food industry and alters the sugar composition of honey by increasing glucose content [32]. Date syrup, which is abundant in the Middle East, has a sugar composition very similar to that of honey, containing approximately 39.4% fructose and 38.2% glucose [33]. The prevalence and compositional similarities of these syrups to honey highlight their relevance as adulterants in authenticity studies.

**Figure 1:**
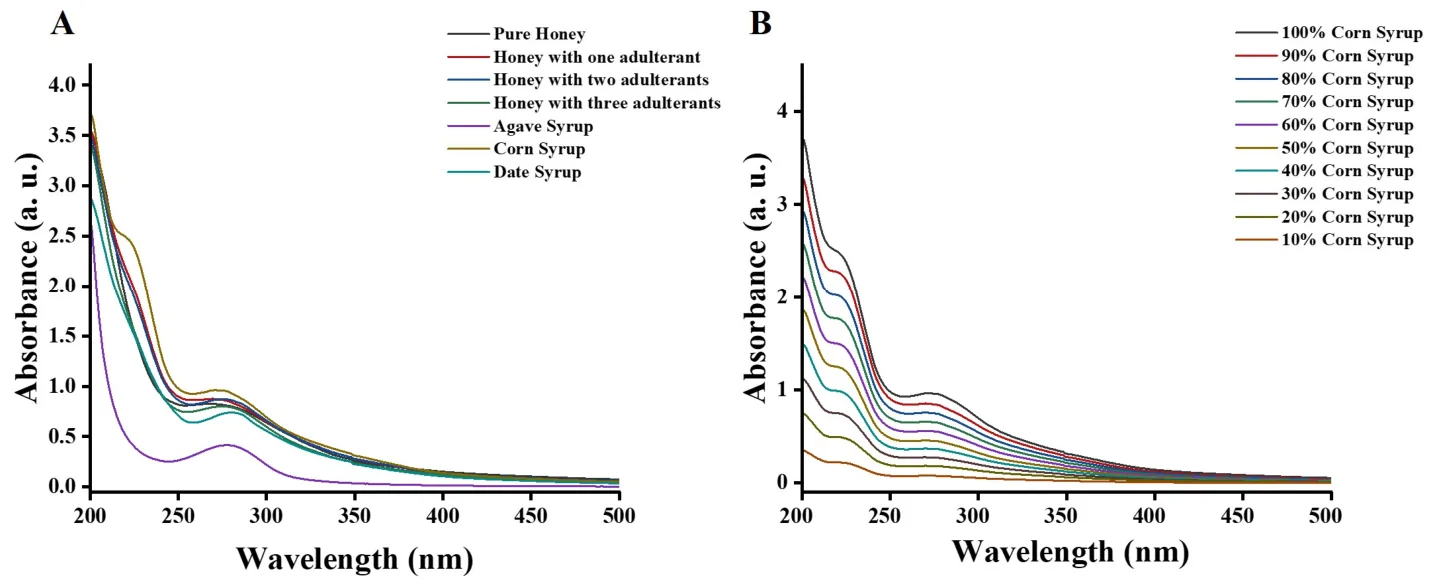
(**A**) Averaged UV–vis spectra of pure honey, honey experimentally adulterated with different syrups, and pure syrup samples; and (**B**) averaged UV–vis spectra of honey adulterated with different concentrations of corn syrup after subtraction of the spectral contribution of pure honey.

All honey samples, including 44 pure Sidr honey samples and 1056 samples experimentally adulterated with corn, agave, and/or date syrups, underwent UV–vis spectroscopic analysis, as depicted in Figure S1. Although high absorbance values were observed in the 200–215 nm region, this spectral range was excluded from chemometric analysis due to potential nonlinearity effects and instrumental artifacts that can be associated with high absorbance. Therefore, only the 215–350 nm region was selected for subsequent multivariate data analysis and model development. The UV–vis spectra for pure honey, individual syrups, and their various mixtures revealed molecular absorption bands in the 215–350 nm region (Figure 1A). Absorption bands within the 215–240 nm range are primarily attributed to sugars, especially glucose and fructose, as well as phenolic compounds, while those within the 260–300 nm range correspond to amino acids (mainly tryptophan), phenolics, and flavonoids [24,34,35]. As shown in Figure 1A, these absorption bands exhibited substantial spectral overlap, particularly in adulterated honey samples. This phenomenon was attributed to the syrups and honey having similar compositions, primarily comprising glucose and fructose, which made their differentiation challenging. Therefore, the spectrum of pure honey was quantitatively subtracted from that of honey containing corn syrup, as illustrated in Figure 1B. Following subtraction, the characteristic absorption bands of corn syrup became observable, and their intensities proportionally increased with increasing corn syrup concentration. This simple approach was effective for binary mixtures in which honey contained a single known adulterant. In practical applications, however, honey may contain one or more unknown adulterants, necessitating the use of more advanced statistical methods. Accordingly, chemometric analysis was employed to discriminate between adulterated and pure honey and quantitatively determine the adulteration content.

### 3.2. Chemometric Analysis

#### 3.2.1. PCA and Loading Scores

**Figure 2:**
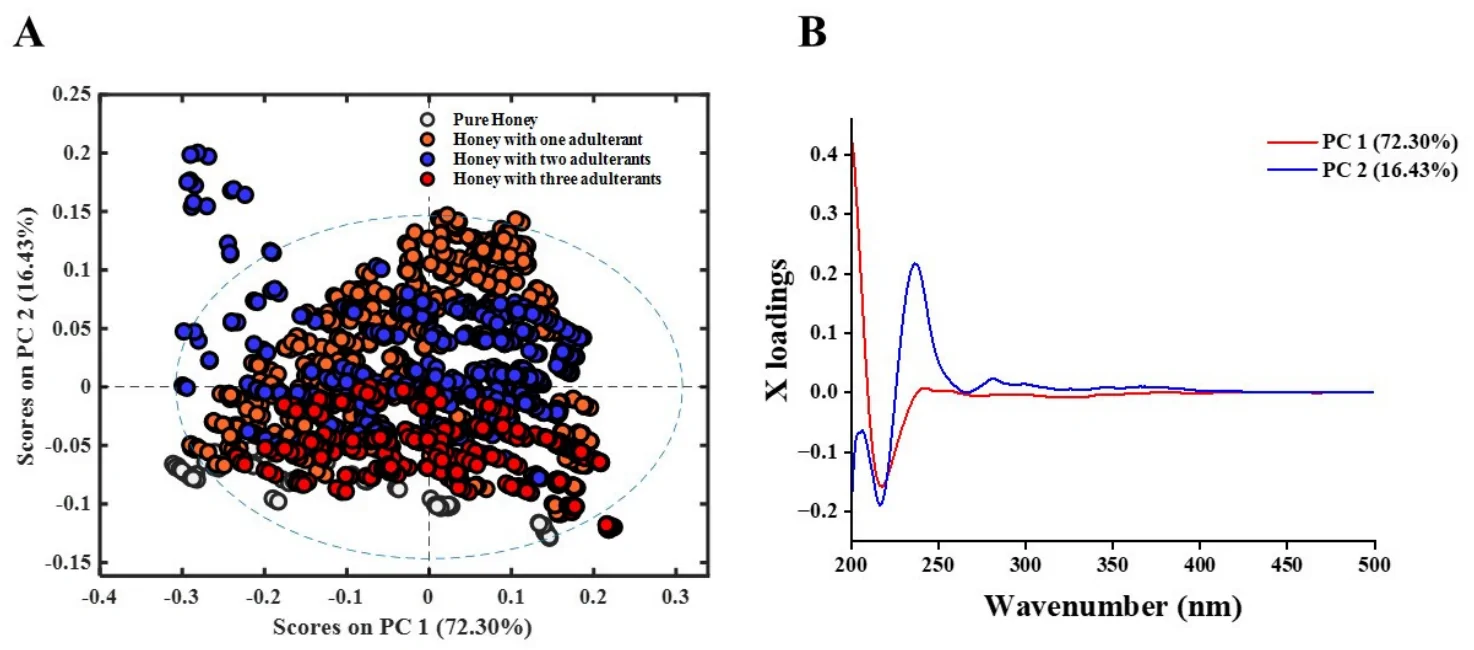
(**A**) Scatter plots for the first two principal components and (**B**) loading plots of the first two components.

An initial exploratory analysis was performed using PCA to investigate whether pure and adulterated honey samples could be visually separated. Figure 2A displays scatter plots of the first two principal components (PCs), which explained approximately 88% of the total variance in the dataset. The PCA plot did not exhibit an evident trend or distinct separation between pure and adulterated honey samples, reflecting the subtle spectral differences between groups.

Further examination of the loading plots identified wavelengths at approximately 215, 236, and 280 nm as being the most informative for distinguishing adulterated samples from pure honey samples (Figure 2B). PC 1 and PC 2 exhibited strong negative loadings at 215 nm, while PC 2 exhibited positive loadings at 236 and 280 nm. Based on these features, classification and regression models were developed to analyze several spectral regions: 215–350 nm (capturing all biomolecules), 215–260 nm (primarily sugars, such as glucose and fructose, and phenolic compounds), and 260–350 nm (amino acids, phenolics, and flavonoids). Other spectral ranges did not provide satisfactory discrimination between pure and adulterated honey samples.

#### 3.2.2. OPLS-DA

OPLS-DA models were employed to effectively distinguish pure honey from adulterated samples and to differentiate between honey samples containing a single adulterant and those containing multiple adulterants. A hierarchical strategy was utilized: first, the presence of an adulterant in a sample was determined; second, whether the sample contained a single or multiple adulterants was determined; and third, the amount of adulterant present in the sample was determined. This multistep analytical workflow provides a comprehensive framework for authenticating honey and monitoring its quality.

**Figure 3:**
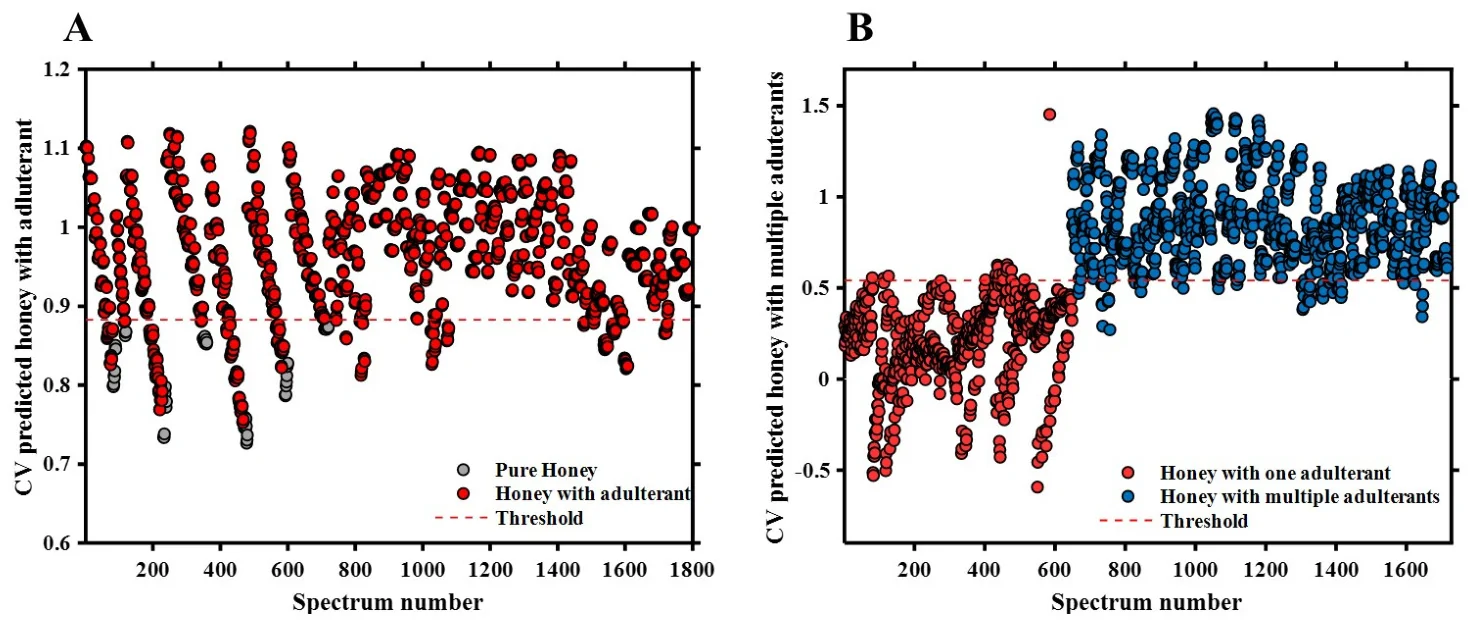
OPLS-DA score plots developed using the 215–260 nm spectral region for the discrimination of (**A**) pure and adulterated honey samples and (**B**) honey samples containing a single adulterant from those containing multiple adulterants. The dashed red line represents the classification threshold.

As described above, three UV–vis spectral regions (251–350, 215–260, and 260–350 nm) were evaluated to develop classification models and their performance was evaluated using RSCV, yielding cross-validation accuracies of 93–95% in distinguishing pure honey from adulterated samples. Figure 3A,B presents representative OPLS-DA score plots for the 215–260 nm spectral region, demonstrating clear discrimination between pure and adulterated honey samples (Figure 3A), as well as between honey samples containing a single adulterant and those containing multiple adulterants (Figure 3B). As shown in Figure 3A, all pure honey spectra were correctly classified, although a limited number of adulterated honey spectra overlapped with the pure honey class. Nevertheless, the model achieved an overall cross-validation prediction accuracy of 94%. Furthermore, excellent separation was achieved between samples containing a single adulterant and those containing multiple adulterants (Figure 3B), with an overall classification accuracy of 96%. Detailed classification performance metrics and confusion matrices for all evaluated spectral regions are provided in Table 2 and Table 3.

Furthermore, an external validation dataset was employed to assess the predictive performance of the models on new, unseen data and their ability to generalize across different data. This was performed using an independent external validation dataset that was not involved in model training. In total, 1500 spectra were uploaded for external validation. Table 2 summarizes the prediction outcomes for cross-validation and external validation. Using the 215–350 nm region, the OPLS-DA model correctly classified 1391 adulterated spectra and 48 pure honey spectra, resulting in an overall accuracy of 88%. A comparable accuracy (88%) was obtained using the 215–260 nm region. However, the predictive performance of the model substantially declined when using the 260–350 nm region, yielding an overall accuracy of only 68% on the external validation dataset. Results indicated that the 215–350 and 215–260 nm spectral regions contained the most informative features for distinguishing pure honey from adulterated samples, whereas the 260–350 nm region provided limited discriminatory power.

Subsequently, three OPLS-DA models were developed to distinguish honey samples containing a single adulterant from those containing multiple adulterants using the UV–vis spectral regions described above. The RSCV results demonstrated high classification accuracy (95–96%) in distinguishing sample classes. Model robustness was further evaluated using an external validation dataset comprising 1400 spectra. The performance of the OPLS-DA models for each spectral region is summarized in Table 3. Using the 215–350 nm region, the model correctly classified 470 out of 540 spectra as honey containing a single adulterant and 848 out of 900 spectra as honey containing multiple adulterants, yielding an overall accuracy of 91%. Similarly, using the 215–260 nm region yielded 513 out of 540 correctly classified spectra as honey containing a single adulterant and 824 out of 900 correctly classified spectra as honey containing multiple adulterants, achieving an overall accuracy of 93%. Using the 260–350 nm region also yielded an accuracy of 93% in distinguishing sample classes. Results demonstrated that all three spectral regions could be used for discriminating honey containing a single adulterant from that containing multiple adulterants.

Notably, the number of adulterated honey samples was substantially larger than that of pure honey samples owing to the use of multiple adulteration ratios for each pure honey sample, as outlined in Table 1. Such class imbalance can bias traditional accuracy-based evaluations; therefore, class-sensitive performance metrics were employed. The F1 score and Matthews correlation coefficient (MCC) are particularly appropriate for imbalanced datasets because they incorporate information from false positives and negatives rather than solely relying on overall accuracy. The former provides a harmonic balance between precision and recall, while the latter offers a correlation-based measure of agreement between predicted and true classes and remains robust under unequal class distributions [36]. For the initial classification model (pure vs. adulterated honey), the macro-averaged F1-score reached approximately 0.80, indicating overall good classification performance across both classes. The corresponding MCC value of 0.61 suggested moderate agreement between predicted and true classifications despite class imbalance. Notably, the pure honey class achieved an F1-score of 0.65, indicating moderate classification performance for the minority class (Table S2). The comparatively lower F1-score observed for the pure honey class may be partially attributed to class imbalance and the limited number of authentic samples available for model training. Furthermore, the false discovery rate for pure honey was approximately 50% at the individual spectrum level. However, evaluation at the sample level provided additional context for these results. For example, using the 215–350 nm spectral region, the 12 misclassified spectra out of the 60 spectra obtained from the pure honey class originated from one honey sample, with all spectra from its jars being misclassified. Therefore, at the sample level, 4 out of 5 pure honey samples were correctly classified, corresponding to 80% accuracy. For the adulterated honey, 48 spectra were misclassified as pure. Of these, 36 spectra originated from two samples (four jars) containing a single adulterant, 3 spectra originated from one jar of one honey sample, and 9 spectra originated from three jars of honey adulterated with multiple adulterants. At the sample level, 117 out of 120 adulterated honey samples were correctly classified, corresponding to 98% accuracy. Nevertheless, these findings should be considered a proof-of-concept, and further validation using a larger number of independent honey samples is required to assess the robustness and broader applicability of the proposed approach. By contrast, when evaluating honey containing a single adulterant versus that containing multiple adulterants, the model achieved an F1 score of approximately 0.93 across adulteration classes and an MCC value of 0.85, suggesting strong and balanced classification performance.

#### 3.2.3. OPLS-R

**Figure 4:**
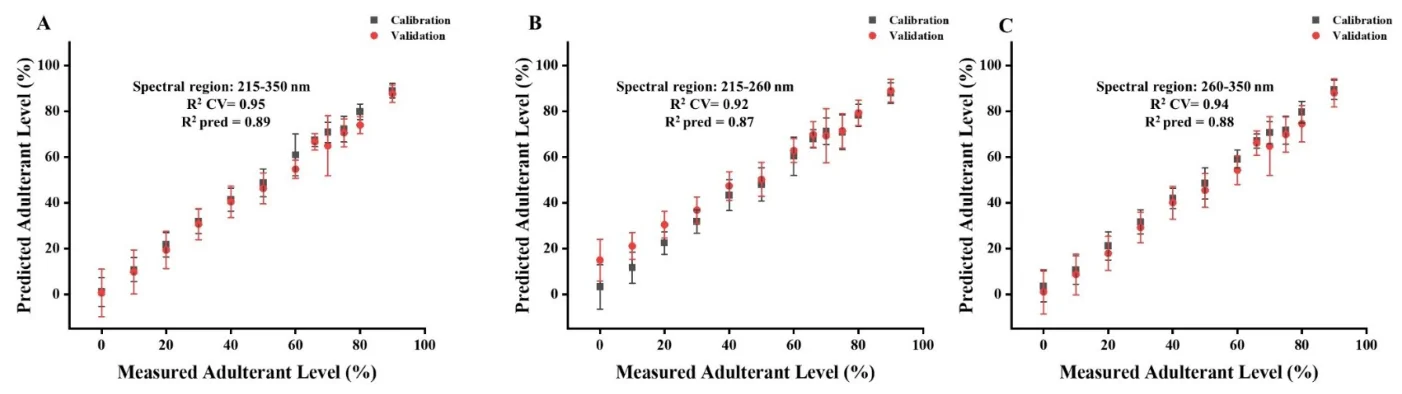
Scatter plots for orthogonal partial least squares (OPLS) regression analysis of adulterated honey samples, displaying average predicted adulterant content versus average measured (actual) value. Predictions were generated using spectral regions: (**A**) 215–350 nm, (**B**) 215–260 nm, and (**C**) 260–350 nm. Error bars represent one standard deviation.

The UV–vis spectral data were subjected to OPLS-R analysis to quantify the adulterant content of each sample. This approach established the relationship between the independent variables (UV–vis spectral features) and dependent variable (adulterant concentration). Figure 4A–C present scatter plots comparing mean predicted adulterant concentrations with experimentally measured values across the UV–vis spectral regions of 215–350, 215–260, and 260–350 nm. Model performance was systematically assessed using standard validation metrics, including the root mean square error of calibration (RMSEC), root mean square error of cross-validation (RMSECV), root mean square error of prediction (RMSEP), and coefficient of determination (R^2^), as summarized in Table 4, providing a comprehensive evaluation of calibration quality, model robustness, and predictive capability. Across all spectral regions, the models exhibited strong calibration and cross-validation performance, with RMSEC and RMSECV values ranging from 5.2% to 6.8% and from 5.5% to 6.9%, respectively, indicating minimal overfitting. The prediction errors of the models were moderately higher, with RMSEP values ranging between 8.4% and 9.2%, reflecting the increased complexity of predicting external samples. High R^2^ values were obtained for calibration and cross-validation (0.92–0.95) and satisfactory R^2^ values were achieved during external validation (0.87–0.89), confirming the reliability and generalizability of the models.

Among the spectral regions analyzed, the 215–350 nm range consistently delivered the best regression performance, as evidenced by lower error metrics and higher R^2^ values during calibration and validation. This spectral interval encompassed the full UV–vis absorption profile of all biomolecular constituents in honey, including sugars, amino acids, and phenols, facilitating more reliable honey authentication. Although the additional wavelengths above 260 nm contributed relatively little to class discrimination, as demonstrated by the comparable performance of the OPLS-DA models using the 215–260 nm and 215–350 nm regions, incorporating the full 215–350 nm spectral range improved the performance of the OPLS-R models, resulting in higher R^2^ values and lower prediction errors during both calibration and external validation.

Spectroscopic techniques such as nuclear magnetic resonance, UV–vis absorption, Fourier-transform infrared (FTIR), Raman, NIR, and mid-infrared spectroscopies have gained substantial attention for the detection of honey adulteration. These methods provide rapid, non-destructive analysis with minimal sample preparation, enabling efficient and practical screening of honey authenticity. Nevertheless, such spectroscopic methods may lack sufficient specificity or sensitivity to detect adulteration when employed as standalone techniques. Consequently, their integration with ML algorithms has become a frequently used strategy for robust identification of honey adulteration [37,38]. Hyperspectral imaging (HSI) has emerged as a promising technique for food quality assessment and honey adulteration detection because it combines both spectral and spatial information, providing a more comprehensive characterization of the sample than conventional spectroscopy. Ahmed [39] employed hyperspectral imaging combined with several machine learning algorithms to detect honey adulteration and reported classification accuracies exceeding 98%, with artificial neural networks (ANNs) providing the best performance. However, the majority of misclassifications occurred for samples containing low adulteration levels (5–10%), particularly for Clover honey, highlighting the challenges associated with detecting subtle compositional changes at low levels of adulteration. Despite its excellent analytical performance, HSI requires specialized instrumentation, large datasets, and more complex data processing than conventional spectroscopy, which may limit its routine implementation.

Front-face fluorescence spectroscopy has also been investigated for honey authentication and adulteration detection. Ropciuc et al. [40] employed excitation–emission matrix (EEM) fluorescence spectroscopy combined with chemometric analysis and successfully differentiated authentic and adulterated honey while simultaneously discriminating between different botanical origins. Among the evaluated machine learning algorithms, SVM outperformed PLS-DA, and classification performance improved with increasing adulteration levels.

Attenuated reflectance (ATR)-FTIR spectroscopy combined with multivariate analysis has shown considerable promise in this context. For example, Dumancas et al. [41] reported optimal performance for detecting adulterated Manuka honey using ATR-FTIR spectroscopy coupled with PLS regression, focusing on the 1501–799 cm^−1^ spectral region. This targeted spectral region resulted in lower RMSECV and RMSEP values and an improved R^2^ value (0.859) compared with models constructed using the full spectral range (R^2^ = 0.859 and RMSEP = 0.014). In another study, Limm et al. [42] combined SIMCA modeling with ATR-FTIR to detect adulterated clover honey in the United States, achieving correct classification rates of 97.6% and 94.8% for adulteration with >7% (*w*/*w*) rice and corn syrups, respectively.

Additionally, Raman spectroscopy combined with chemometrics has demonstrated strong potential for honey authentication. Support vector machine (SVM), probabilistic neural network (PNN), and convolutional neural network (CNN) models were successfully coupled with Raman spectroscopy to detect Zhejiang Suichang native honey adulterated with maltose syrup, enabling the detection of low-level adulteration with high classification accuracy [43]. Moreover, UV–vis absorption spectroscopy has provided a valuable analytical tool for honey authentication. In two studies, DD-SIMCA models combined with UV–vis spectroscopy enabled the detection of a wide range of adulterants, including corn and agave syrups, sugarcane molasses, various sugar syrups, and added colorants [23,24]. Furthermore, Valinger et al. [26] combined UV–vis and NIR spectroscopy with PLS and ANN modeling to detect and quantify the physicochemical properties of pure and adulterated honey samples. ANN models provided accurate and simultaneous predictions, with validation R^2^ values exceeding 0.8, underscoring the strong predictive capability of neural-network-based approaches for honey authentication.

The present study expands upon previous research by integrating UV–vis absorption spectroscopy and ML algorithms for qualitative and quantitative detection of honey adulteration. OPLS-DA models were successfully employed to identify the presence of corn, agave, and date syrups in locally produced Kuwaiti Sidr honey. Despite the high commercial value and regional importance of Kuwaiti Sidr honey, authenticity studies have predominantly employed sophisticated analytical techniques, including mass spectrometry-based methods, which are often time-consuming and require extensive sample preparation [5,28]. In particular, studies evaluating multiple adulterants and mixed-adulterant systems remain limited [23]. In the present work, OPLS-DA models were employed to detect adulteration of locally produced Kuwaiti Sidr honey with corn, agave, and date syrups. The models also enabled discrimination between samples containing a single adulterant and those containing multiple adulterants. In addition, OPLS-R analysis enabled accurate quantification of adulterant content, regardless of whether one or multiple adulterants were present in the sample, with relatively low prediction error. The ability to both detect and quantify adulteration in mixed-adulterant systems represents an advancement over previously reported approaches that focused primarily on classification or single-adulterant models [5,24]. The inclusion of binary, ternary, and quaternary mixtures extends the evaluation beyond single-adulterant systems to more realistic fraud scenarios, where multiple adulterants may be combined to mask adulteration and complicate authenticity assessment [44]. Collectively, results demonstrated that the proposed approach provides a simple and sensitive tool for detecting and quantifying honey adulteration. Moreover, the approach offers several practical advantages, including minimal sample preparation (requiring only simple dilution), rapid data acquisition, and cost-effectiveness, enabling routine implementation without the need for complex or expensive analytical procedures.

Although the results obtained in this study are promising, the models were developed and validated specifically using Kuwaiti Sidr honey samples. Differences in botanical origin, geographical provenance, and compositional profiles may affect spectral characteristics, potentially limiting direct applicability to other honey types. Nevertheless, the same analytical framework can be readily extended to other honeys and adulterants through model retraining and optimization. This may include expanding calibration datasets, tuning chemometric parameters, and incorporating additional validation samples to ensure robust and generalized performance across diverse honey matrices. In addition, the present study was performed using honey samples analyzed under controlled laboratory conditions. Factors encountered during routine analysis, including long-term storage, natural crystallization, and instrumental variability, may influence the UV–Vis spectra and, consequently, the model performance. Prolonged storage may alter the chemical composition of honey through processes such as Maillard reactions, oxidation, and degradation of phenolic compounds, leading to subtle changes in the absorption spectra. Furthermore, natural crystallization can modify light scattering properties and sample homogeneity, potentially affecting absorbance measurements if samples are not adequately homogenized prior to analysis [45]. Additionally, instrument-to-instrument variability, including differences in spectral resolution, detector response, and optical configuration, may introduce systematic spectral variations that could influence model performance. Therefore, future studies will evaluate honey samples subjected to different storage conditions, varying degrees of crystallization, and measurements acquired on multiple UV–Vis instruments. Incorporating these sources of variability into the calibration dataset is expected to improve model robustness and facilitate routine implementation across different laboratories.

## 4. Conclusions

This study proof-of-concept study highlights the use of UV–vis absorption spectroscopy combined with ML algorithms for the qualitative and quantitative detection of honey adulteration. Pure honey and samples experimentally adulterated with corn, agave, and date syrups were analyzed following simple dilution in water, and UV–vis spectra were collected within the 200–500 nm range. Initially, PCA was employed to identify three UV spectral regions that most strongly contributed to spectral variation, thereby identifying the most informative wavelength ranges. Using these three spectral regions, OPLS-DA models classified pure versus adulterated honey with 88% accuracy on the external validation dataset, and a second set of OPLS-DA models further differentiated between samples containing a single adulterant and those containing multiple adulterants with 93% accuracy on external validation samples. Quantitative estimation of adulterant content was performed via OPLS-R analysis, which yielded satisfactory predictive performance on external validation samples (R^2^ ≥ 87). Collectively, these results demonstrate the potential of the proposed approach as a cost-effective and non-destructive screening tool for honey authentication, while supporting its application as a preliminary proof-of-concept study. The method requires minimal sample preparation, avoids labor-intensive procedures, and offers practical advantages for routine implementation by regulatory agencies, producers, and consumers to reduce honey fraud and ensure product integrity.

The developed models should be regarded as a proof-of-concept framework for detecting adulteration in Kuwaiti Sidr honey under controlled experimental conditions. Before routine practical implementation, further validation using independently sourced commercial honey samples from broader botanical and geographical origins is required. Future studies will therefore expand the calibration and validation datasets to include Sidr honey from neighboring countries, other honey varieties, lower adulteration levels, commercially adulterated samples, and more complex or unknown adulteration scenarios. Such samples may exhibit greater compositional and spectral variability because of differences in processing, storage, adulterant type, and natural honey heterogeneity. Incorporating these sources of variability will provide a more rigorous assessment of model robustness and real-world applicability. In addition, successful implementation of this approach could support the establishment of a centralized honey authentication service or reference laboratory, where honey samples submitted by producers, regulatory agencies, and industry stakeholders could be routinely analyzed. Such a facility would continuously expand the spectral database by incorporating newly marketed honey products, additional geographical origins, and emerging adulteration practices, while periodically retraining and independently validating the chemometric models as new data become available. In this way, the proposed UV–Vis spectroscopy and chemometric framework should be viewed as an adaptive analytical platform rather than a static model, with its predictive performance improving continuously as the reference database grows. This strategy would facilitate long-term implementation in routine quality control and regulatory monitoring and support the development of a national reference resource for honey authentication.

## Figures and Tables

**Table 1:** Experimentally adulterated samples prepared with honey (H), corn syrup (C), date syrup (D), and agave syrup (A).

Binary Mixture (Honey/One Adulterant; %*v*/*v*) H/C	Ternary Mixture (Honey/Two Adulterants; %*v*/*v*) H/C/D	Quaternary Mixture (Honey/Three Adulterants; %*v*/*v*) H/C/D/A
**10/90**	34/33/33	50/10/10/30
**20/80**	25/25/50	50/30/10/10
**30/70**	50/25/25	50/10/30/10
**40/60**	25/50/25	70/10/10/10
**50/50**	10/30/60	25/25/25/25
**60/40**	30/10/60	-
**70/30**	30/60/10	-
**80/20**	60/10/30	-
**90/10**	60/30/10	-
	10/60/30	-

**Table 2:** Confusion matrices for cross-validation and external validation of OPLS-DA models based on different spectral regions for pure and adulterated honey.

**Spectral region 215–350 nm LV = 2**				
**Predicted as**	Internal cross-validation results		External validation results	
	Pure Honey	Adulterated Honey	Pure Honey	Adulterated Honey
**Pure Honey**	72	245	48	48
**Adulterated Honey**	0	1483	12	1392
**Unassigned**	0	0	0	0
**Prediction rate**	93%		88%	
**Spectral region 215–260 nm LV = 3**				
**Predicted as**	Internal cross-validation results		External validation results	
	Pure Honey	Adulterated Honey	Pure Honey	Adulterated Honey
**Pure Honey**	72	216	48	49
**Adulterated Honey**	0	1512	12	1391
**Unassigned**	0	0	0	0
**Prediction rate**	94%		88%	
**Spectral region 260–350 nm LV = 6**				
**Predicted as**	Internal cross-validation results		External validation results	
	Pure Honey	Adulterated Honey	Pure Honey	Adulterated Honey
**Pure Honey**	70	135	24	59
**Adulterated Honey**	2	1593	36	1381
**Unassigned**	0	0		
**Prediction rate**	95%		68%	

**Table 3:** Confusion matrices for cross-validation and external validation of OPLS-DA models for honey containing single and multiple adulterants.

**Spectral region 215–350 nm LV = 10**				
**Predicted as**	Internal cross-validation results		External validation results	
	Honey with one adulterant	Honey with multiple adulterants	Honey with one adulterant	Honey with multiple adulterants
**Honey with one adulterant**	619	52	470	52
**Honey with multiple adulterants**	29	1028	70	848
**Unassigned**	0	0	0	0
**Prediction rate**	96%		91%	
**Spectral region 215–260 nm LV = 10**				
**Predicted as**	Internal cross-validation results		External validation results	
	Honey with one adulterant	Honey with multiple adulterants	Honey with one adulterant	Honey with multiple adulterants
**Honey with one adulterant**	628	59	513	76
**Honey with multiple adulterants**	20	1021	27	824
**Unassigned**	0	0	0	0
**Prediction rate**	96%		93%	
**Spectral region 260–350 nm LV = 11**				
**Predicted as**	Internal cross-validation results		External validation results	
	Honey with one adulterant	Honey with multiple adulterants	Honey with one adulterant	Honey with multiple adulterants
**Honey with one adulterant**	617	50	471	11
**Honey with multiple adulterants**	31	1030	69	889
**Unassigned**	0	0	0	0
**Prediction rate**	95%		93%	

**Table 4:** Standard validation metrics for OPLS-R analysis of honey adulteration.

Parameter	Spectra Region 215–350 nm	Spectral Region 215–260 nm	Spectral Region 260–350 nm
RMSEC	5.2	6.8	5.7
RMSECV	5.5	7.0	5.9
REMSEP	8.4	9.2	9.0
R^2^ Cal	0.95	0.92	0.94
R^2^ CV	0.95	0.92	0.94
R^2^ Pred	0.89	0.87	0.88

## Data Availability

The original contributions presented in this study are included in the article/Supplementary Material. Further inquiries can be directed to the corresponding authors.

## Supplementary Materials

The following supporting information can be downloaded at https://www.mdpi.com/article/10.3390/foods15172953/s1, Figure S1: UV-Vis spectra of pure honey and adulterated honey with corn, date, and agave syrups; Table S1: Summarizes the honey source, associated dollar amount, and whether each sample was used for model training or validation; Table S2: Class-wise precision, recall (TPR), and F1-score values obtained for the classification models used to detect honey adulteration.
