# Supplementary material for: Detection of Honey Adulteration Using UV–Vis Absorption Spectroscopy and Chemometrics
Source: Foods. 2026 Aug 22;15(17):2953. doi: 10.3390/foods15172953 (PMC13565015; doi:10.3390/foods15172953)
Supplement: Supplementary file 1 [file foods-15-02953-s001.zip › foods-4414363-supplementary.pdf]

Supplementary Material

Detection of Honey Adulteration using UV–Vis Absorption Spectroscopy and Chemometrics

Aya Ibrahim[a#], Mohamed O. Amin[b#], Bessy D`Cruz[a], Bhavik Vyas[b], Igor K. Lednev[b*], Entesar Al-Hetlani[a*]

[a] Department of Chemistry, College of Science, Kuwait University, Sabah Al Salem University City, P.O. Box 5969, Safat 13060, Shadadiya, Kuwait

[b] Department of Chemistry, University at Albany, SUNY, 1400 Washington Avenue, Albany, NY 12222, United States

Corresponding authors:

Igor K. Lednev: ilednev@albany.edu

Entesar Al-Hetlani: entesar.alhetlani@ku.edu.kw

Table S1. Summarizes the honey source, associated dollar amount, and whether each sample was used for model training or validation.

Honey code	Source	Cost per 100 gram (USD/100 g)	Cal/Val
**S1**	Supermarket – south	7	Cal
**S2**	Direct producer- south	8	Cal
**S3**	Direct producer- south	12	Val
**S4**	Direct producer- south	13	Cal
**S5**	Direct producer- south	8	Cal
**S6**	Direct producer- south	8	Val
**N1**	Supermarket – north	7	Val
**N2**	Direct producer- north	11	Cal
**N3**	Direct producer- north	5	Val
**H1**	Direct producer	17	Cal
**H2**	Direct producer	17	Val

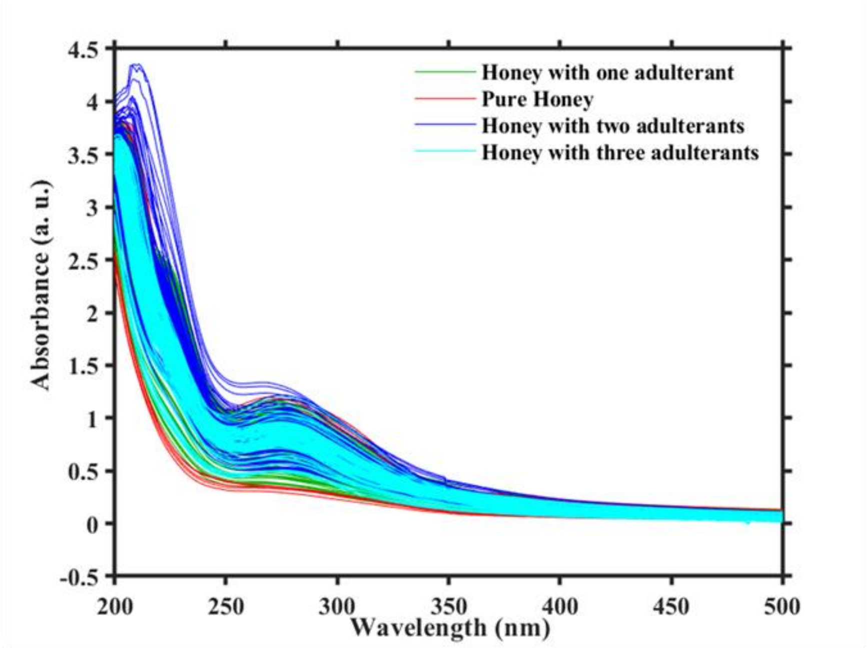

Figure S1: UV-Vis spectra of pure honey and adulterated honey with corn, date, and agave syrups.

Table S2: Class-wise precision, recall (TPR), and F1-score values obtained for the classification models used to detect honey adulteration.

Class	Precision	Recall (TPR)	F1-score
**Pure Honey**	0.552	0.800	0.653
**Honey with adulterant**	0.977	0.928	0.952
**Honey with one adulterant**	0.871	0.950	0.909
**Honey with two/three adulterants**	0.968	0.916	0.941
